# Long-Standing, Giant, Right-Sided Inguinal Hernia Containing the Right Colon and Greater Omentum: A Case Report

**DOI:** 10.7759/cureus.93169

**Published:** 2025-09-25

**Authors:** Bartosz Czyzewski, Karol Klosinski, Joanna Czyzewska, Alicja Dorota, Mateusz Jeckowski

**Affiliations:** 1 Student Scientific Club of Biomedicine and Experimental Surgery, Faculty of Medicine, Medical University of Lodz, Łódź, POL; 2 Department of Biomedicine and Experimental Surgery, Faculty of Medicine, Medical University of Lodz, Łódź, POL; 3 Medical School, Zaglebiowskie Oncology Center in Dąbrowa Górnicza, Dąbrowa Górnicza, POL; 4 Department of Oncological Surgery, Nicolaus Copernicus Provincial Multispecialty Center for Oncology and Traumatology in Lodz, Łódź, POL

**Keywords:** case report, giant inguinal hernia, greater omentum resection, lichtenstein repair, respiratory prophylaxis, scrotal hematoma

## Abstract

Giant inguinal hernias are rare, long-standing conditions characterized by massive herniation of intra-abdominal organs into the scrotum. Their chronicity, unusual contents, and associated comorbidities make diagnosis and surgical management particularly challenging. We report the case of a 63-year-old man with a right-sided inguinal hernia present for 39 years. His medical history included hypertension, ischemic heart disease, hypercholesterolemia, and diabetes mellitus. Preoperative assessment included recommendations for respiratory rehabilitation to optimize pulmonary function. During surgery, the hernia sac was found to contain the right colon, transverse colon, and greater omentum. Resection of the greater omentum was performed, followed by reduction of the hernia contents and repair using the Lichtenstein technique. The postoperative course was complicated by a scrotal hematoma, which required drainage and placement of three drains. The patient recovered well following appropriate management. Giant inguinal hernias pose significant intraoperative and postoperative risks, including increased intra-abdominal pressure, respiratory compromise, and hematoma formation. Careful preoperative planning, meticulous surgical technique, and individualized management are crucial for successful outcomes. Preventive strategies such as preoperative respiratory prophylaxis may further reduce postoperative complications. In addition, increasing public awareness is important to encourage earlier presentation, reduce the risk of complications, and minimize stigmatization associated with the condition. Giant inguinal hernias remain a rare but challenging surgical entity. Early diagnosis, timely surgical intervention, and multidisciplinary perioperative care are essential to achieving safe outcomes and improving quality of life.

## Introduction

Inguinal hernia is one of the most common surgical conditions worldwide, accounting for approximately 75% of all abdominal wall hernias, with a lifetime incidence of 27% in men and 3% in women [1]. Most hernias are diagnosed and repaired early; however, in rare cases, they may remain untreated for decades, resulting in massive, long-standing hernias with unusual contents. We present a case of a giant, long-standing inguinal hernia containing gastrointestinal structures and greater omentum, which required surgical treatment consisting of resection and Lichtenstein hernioplasty.

## Case presentation

A 63-year-old man was admitted to the Department of Oncological Surgery with a right-sided inguinal hernia that had been present for 39 years. His medical history included hypertension, ischemic heart disease with an episode of acute coronary syndrome 16 years earlier, hypercholesterolemia, and diabetes mellitus. He had previously undergone surgery consisting of left-sided inguinal hernia repair using the Bassini technique and excision of a penile lesion. His chronic medications included amlodipine/hydrochlorothiazide/valsartan, spironolactone, nebivolol, acetylsalicylic acid, empagliflozin, and atorvastatin. He reported no known drug allergies.

**Figure 1:**
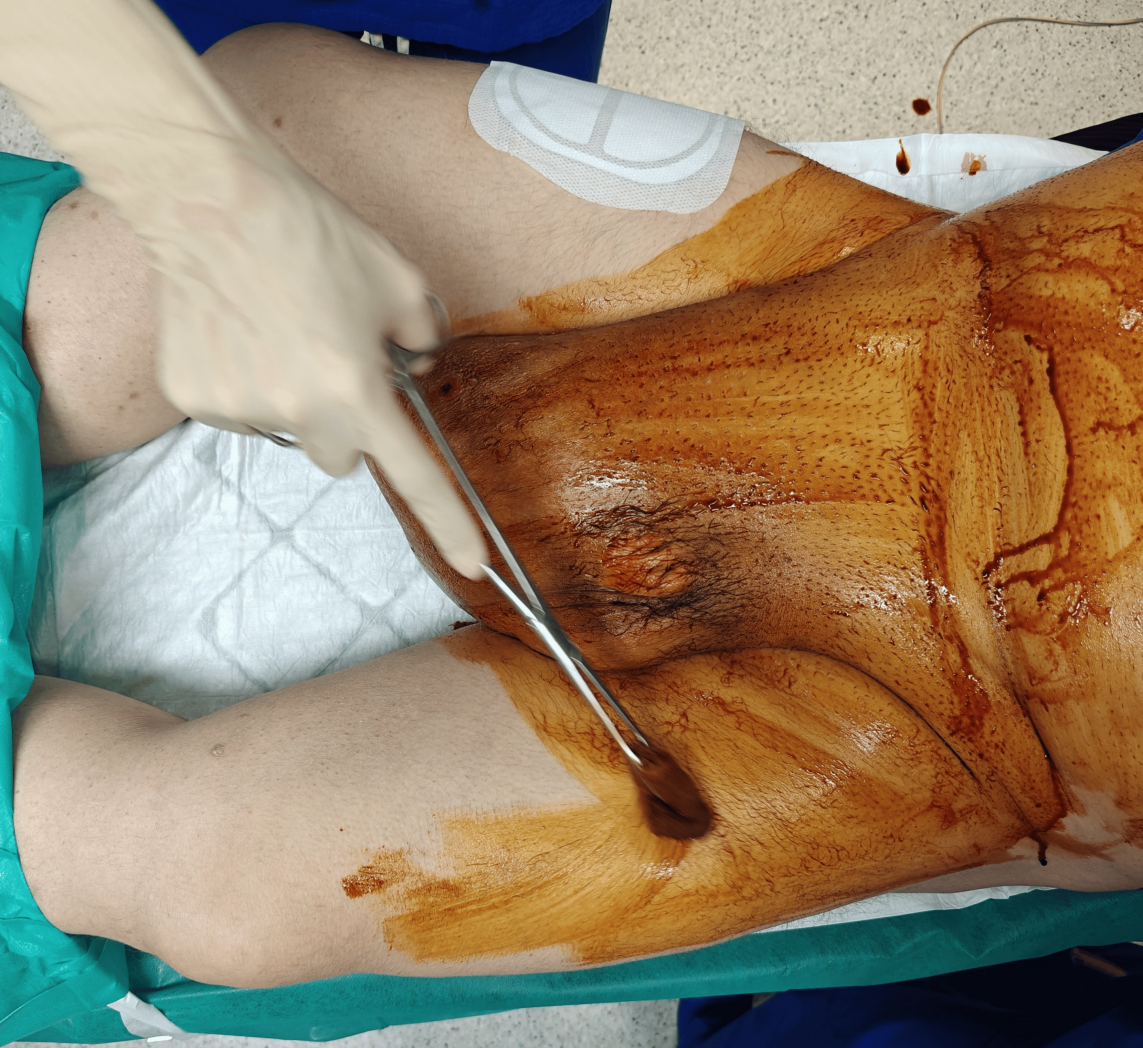
Preoperative view of the right-sided inguinal hernia.

**Figure 2:**
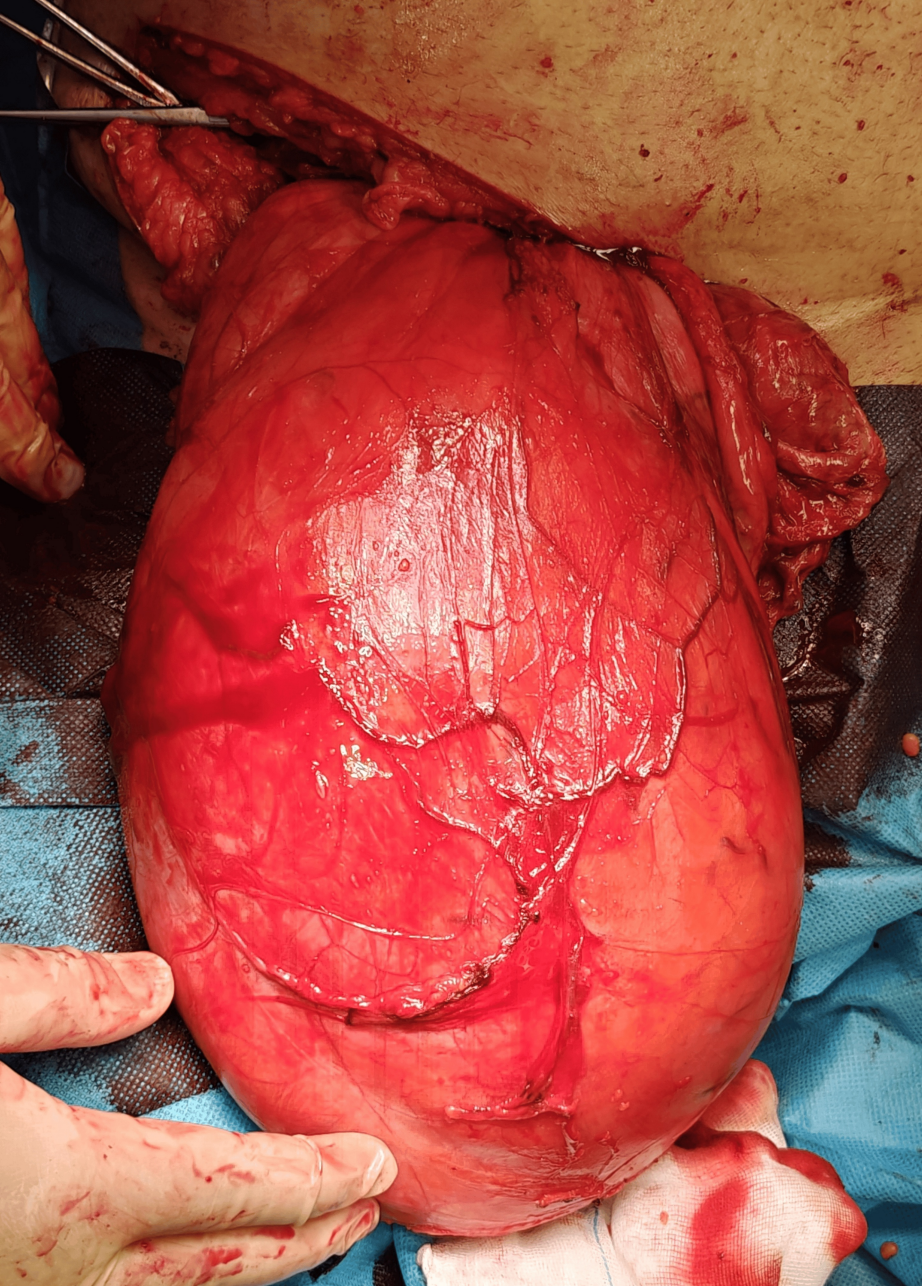
Intraoperative view of the hernia sac after dissection.

**Figure 3:**
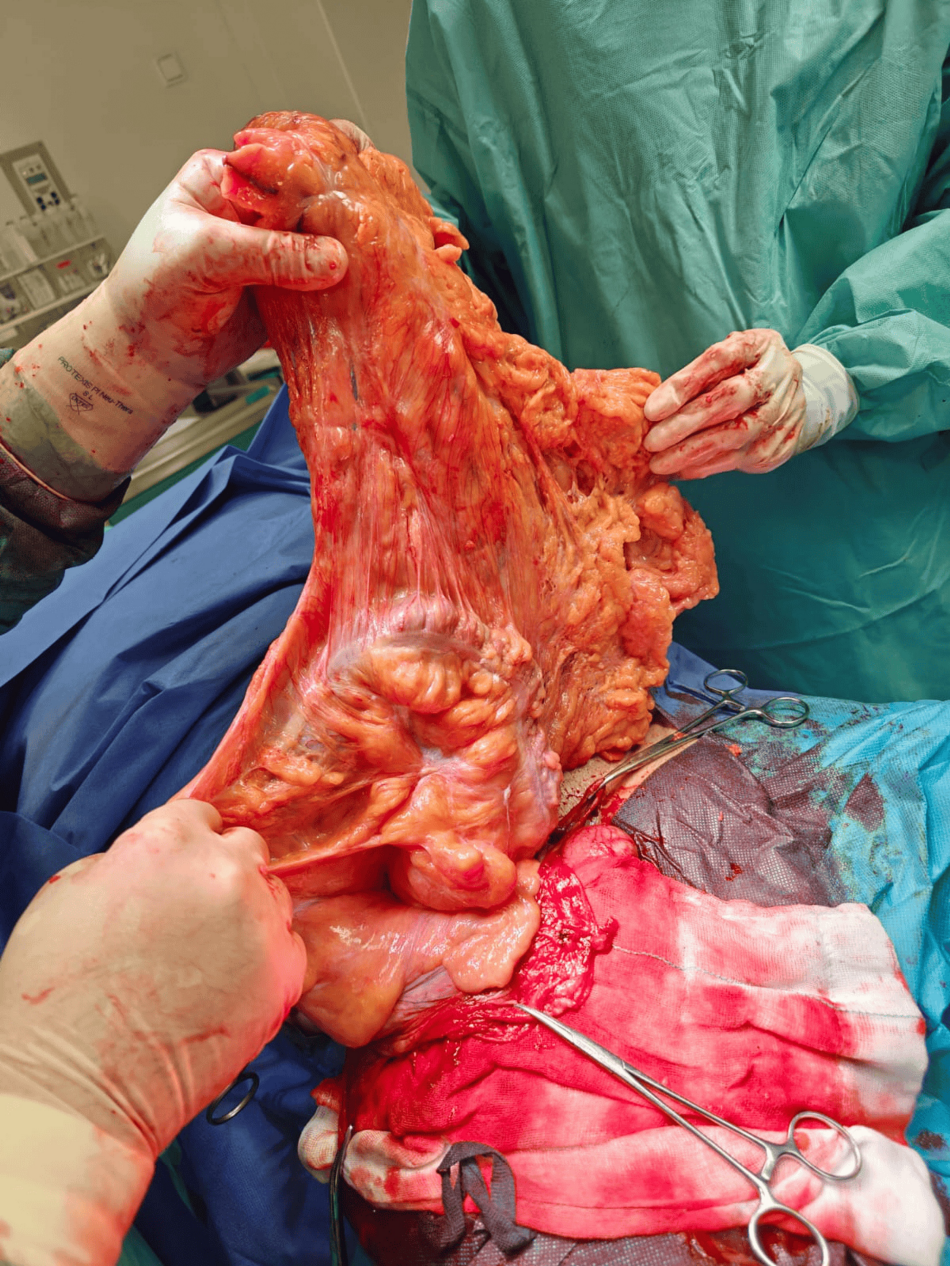
Contents of the hernia sac, including the right colon, transverse colon, and greater omentum.

During hospitalization, the patient underwent unilateral right-sided inguinal hernia repair. Intraoperatively, the hernia sac was found to contain the right colon, transverse colon, and greater omentum (Figures 1-3). Resection of the omentum was performed, the hernia contents were reduced, and the defect was repaired using the Lichtenstein technique with the implantation of an Optilene Mesh 15 × 20 cm. On the fifth postoperative day, a scrotal hematoma developed, which was managed with surgical drainage. Three drains were placed: one in the scrotum, one above the mesh, and one in the subcutaneous tissue.

## Discussion

A giant inguinal hernia is characterized by massive herniation of intra-abdominal organs into the scrotum. Various organs have been reported within giant inguinal hernias, including the appendix, urinary bladder, small and large bowel, stomach, and even ovaries [2]. In the present case, the hernia had been present for 39 years, resulting in marked enlargement of the hernia sac and displacement of the right colon, transverse colon, and greater omentum into the sac. This wide spectrum of potential hernia contents underscores the challenges in both diagnosis and surgical management.

During surgical repair, particular attention should be paid to the risk of increased intra-abdominal pressure following repositioning of the hernia contents. A sudden rise in intra-abdominal pressure can impair regional blood flow, decrease venous return and cardiac output, and compromise respiratory function due to diaphragmatic elevation. These physiological disturbances may contribute to the increased risk of mortality reported after forced reduction. To mitigate these risks, preventive strategies include careful intraoperative decision-making, selective resection of hernia contents (omentum, colon, or small bowel), and, in selected cases, preoperative progressive pneumoperitoneum to gradually increase the abdominal domain [3-5].

In our patient, resection of the greater omentum was performed, followed by reduction of the hernia contents and repair using the Lichtenstein technique. This approach is considered both feasible and safe, providing high patient satisfaction and improved postoperative quality of life [6]. Additionally, the patient was advised to undergo preoperative respiratory rehabilitation to optimize pulmonary function and reduce the risk of postoperative complications.

Postoperative complications of giant inguinal hernia repair include hematoma, ischemic orchitis, surgical site infection, and seroma [7]. Scrotal hematoma is particularly common and is often associated with dense adhesions resulting from the chronicity of the disease. Because closed drainage systems do not always prevent hematoma formation, meticulous intraoperative hemostasis combined with vigilant postoperative monitoring is essential for both prevention and early detection [5].

The long-standing course of the disease, combined with the unusual hernia contents, makes this case particularly noteworthy. It highlights the importance of individualized surgical planning and meticulous intraoperative management to prevent life-threatening complications while optimizing both functional and aesthetic outcomes.

## Conclusions

This case highlights the challenges of managing long-standing, giant inguinal hernias. Careful preoperative planning, meticulous surgical technique, and individualized management are crucial to safely reduce the hernia contents and prevent complications. In this patient, resection of the greater omentum combined with Lichtenstein repair proved to be an effective approach. In addition, preoperative respiratory prophylaxis should be considered to optimize pulmonary function and minimize postoperative complications. Early diagnosis and timely surgical intervention remain essential to avoid the development of such extreme cases. Increasing public awareness is also important to promote earlier detection and treatment, thereby reducing the risk of complications and helping avoid the stigmatization often associated with this condition.

## References

[1] Kingsnorth A, LeBlanc K (2003). Hernias: inguinal and incisional. Lancet.

[2] Creedon L, Peacock O, Singh R, Awan A (2014). Gastric outlet obstruction secondary to incarcerated pylorus in an inguinal hernia. Ann R Coll Surg Engl.

[3] Barst HH (1972). Pneumoperitoneum as an aid in the surgical treatment of giant herniae. Br J Surg.

[4] Papavramidis TS, Marinis AD, Pliakos I, Kesisoglou I, Papavramidou N (2011). Abdominal compartment syndrome - intra-abdominal hypertension: defining, diagnosing, and managing. J Emerg Trauma Shock.

[5] Trakarnsagna A, Chinswangwatanakul V, Methasate A (2014). Giant inguinal hernia: report of a case and reviews of surgical techniques. Int J Surg Case Rep.

[6] Cuihong J, Fan W, Yingmo S (2024). Lichtenstein repair for giant inguinoscrotal hernia: a retrospective case-control study. Hernia.

[7] Demma JA, Gefen R, Shpigelman O, Pikarsky A, Almogy G (2023). Giant inguinal hernia repair using standard transverse inguinal incision with mesh. A retrospective case control study. BMC Surg.

